# Reliability of Malnutrition Assessment Based on Selected Laboratory Parameters in Heart Transplant Recipients—A Retrospective Single-Centre Pilot Study from Poland

**DOI:** 10.3390/nu18010071

**Published:** 2025-12-25

**Authors:** Łukasz J. Krzych, Magdalena Kwiatkowska, Michał Kisiołek, Dominika Krupnik, Bogumiła Król, Piotr Przybyłowski

**Affiliations:** 1 Department of Acute Medicine, Faculty of Medical Sciences in Zabrze, Medical University of Silesia in Katowice, 40-055 Katowice, Poland; 2 Department of Anaesthesiology and Intensive Care, Upper Silesian Medical Centre, 40-752 Katowice, Poland; 3 Students’ Scientific Society “#Intensywna_Po_Godzinach”, Department of Acute Medicine, Medical University of Silesia, 41-800 Zabrze, Poland; 4 Poltransplant Regional Office, 02-001 Warsaw, Poland; 5 Department of Cardiac Surgery, Transplantology, Vascular and Endovascular Surgery, Faculty of Medical Sciences in Zabrze, Medical University of Silesia in Katowice, 40-055 Katowice, Poland

**Keywords:** malnutrition, heart transplant recipients, laboratory biomarkers, retrospective analysis, nutritional status, Poland

## Abstract

**Background:** Malnutrition is a common yet often underestimated risk factor for adverse outcomes in hospitalized patients, including heart transplant recipients. Assessing nutritional status in this population is challenging due to comorbidities, pharmacotherapy, and the urgent nature of surgery. This study aimed to evaluate the reliability of routinely measured laboratory and anthropometric parameters in diagnosing malnutrition in heart transplant patients. **Methods:** This retrospective study included 53 adult patients who underwent orthotopic heart transplantation between 2021 and 2024 at the Silesian Center for Heart Diseases in Zabrze, Poland. Anthropometric data (gender, age, BMI) and laboratory parameters—albumin, total protein, hemoglobin, cholesterol, *C*-reactive protein (CRP), and neutrophil-to-lymphocyte ratio (NLR)—were analyzed. Malnutrition was defined as BMI < 22 kg/m^2^. Cut-off points were based on literature data. Correlations between laboratory parameters and nutritional status were assessed, and concordance in malnutrition classification was evaluated. **Results:** Malnutrition criteria were met by 15% of patients. Only CRP predicted malnutrition risk, though its values did not differ significantly between BMI groups (*p* = 0.106). Negative correlations were found between CRP and total protein (−0.342; *p* = 0.012), albumin (−0.666; *p* < 0.0001), cholesterol (−0.287; *p* = 0.037), and hemoglobin (−0.383; *p* = 0.0046). A positive correlation was observed between CRP and NLR (0.333; *p* = 0.014). **Conclusions:** Malnutrition assessment in heart transplant recipients should not rely solely on individual laboratory parameters. A multifactorial approach integrating biochemical, anthropometric, and clinical data is necessary. Further research is needed to identify novel biomarkers to improve malnutrition risk evaluation and guide nutritional interventions in this population.

## 1. Introduction

Malnutrition is a common, yet underestimated risk factor for poor prognosis in hospitalized patients [1]. Malnutrition affects approximately 20–50% of hospitalized patients [1,2,3]. In the absence of appropriate treatment, up to two-thirds of those already malnourished experience further deterioration during hospitalization, while about one-third of patients admitted in a normal nutritional state develop malnutrition during their hospital stay [4]. Among patients with cardiovascular diseases (CVD), the prevalence of malnutrition reaches up to 70%, depending on the assessment method used. This condition is associated with disease progression, reduced exercise tolerance, increased rates of rehospitalization, and poorer overall prognosis [5].

Optimization of a patient’s health status prior to planned surgery can significantly reduce the risk of postoperative complications [2]. However, such a prehabilitation strategy has limited applicability in two dimensions. The first relates to the urgency of the procedure or the potential for urgency to arise within an unpredictable time frame. In this situation, the limiting factor is time, which may be insufficient to achieve a satisfactory therapeutic effect.

The second aspect concerns comorbidities, the extent to which they are controlled, and their impact on current functioning—factors that can be assessed, for example, using the American Society of Anesthesiologists Physical Status (ASA-PS) classification [3]. In this case, the challenge lies in the patient’s adaptive capacity to undergo nutritional intervention, particularly when combined with the recommended physical activity aimed at improving global metabolism. This issue is particularly relevant to hospitalized older adults, who belong to a high-risk group for adverse health outcomes. Limited physical activity in this population promotes the development of sarcopenia. Frailty syndrome is observed in approximately 40–80% of patients with heart failure (HF), and its prevalence increases with age and disease [6]. Chronic inflammation accompanying HF, characterized by elevated levels of pro-inflammatory cytokines such as TNF-α and interleukin-6, enhances muscle catabolism, thereby contributing to sarcopenia and the development of frailty. Frailty and malnutrition frequently coexist, forming a vicious cycle of mutually reinforcing metabolic and functional impairments. The coexistence of these conditions is associated with poorer clinical outcomes, more frequent rehospitalizations, reduced quality of life, and increased mortality. The shared pathophysiological mechanism underlying these disorders is chronic inflammation, which impairs muscle function, suppresses appetite, and increases protein degradation, thereby contributing to disease progression [7]. Additionally, reduced mobility and impaired muscle coordination hinder chewing and swallowing, significantly limiting the absorption of essential nutrients [8]. Another factor increasing the risk of malnutrition is pharmacotherapy, which may lead to decreased appetite, irritation of the gastrointestinal mucosa, impaired intestinal motility, and reduced saliva production, all of which can adversely affect the patient’s nutritional status. Furthermore, steroid therapy following heart transplantation alters body composition and metabolism, contributing to weight gain, and glucocorticoids may indirectly affect albumin levels by modulating inflammation and protein metabolism [9,10,11]. Special attention should be given to patients with obesity, who may be paradoxically malnourished. This condition, known as sarcopenic obesity, is characterized by a loss of muscle mass accompanied by an excess of adipose tissue [6]. These patients may also experience qualitative malnutrition, defined as deficiencies in vitamins and minerals despite a high BMI.

Additional factors, although difficult to evaluate parametrically, include organizational and administrative aspects of the healthcare system, which are associated with prolonged hospital stays and an increased risk of potentially preventable hospital complications such as pressure ulcers, infections, and surgical site infections [12]. Equally important are patient cooperation, treatment tolerance, and the risk of interactions between medications and nutrients.

It is equally problematic in clinical practice to accurately determine the nutritional status. Currently, there is no unified definition of malnutrition or consistent nutritional guidelines for patients with HF [5], which would take into account both the course of the disease and the presence of comorbidities.

The diagnosis of nutritional disorders is further complicated by the lack of an ideal tool for assessing the degree of malnutrition. Nutritional status may be assessed using anthropometric parameters (e.g., body weight, body mass index (BMI), body composition, and their temporal changes) as well as laboratory measures (e.g., prealbumin, transferrin, albumin, total protein, lymphocyte count, and cholesterol concentration) [13,14,15,16,17]. Each of these approaches has significant limitations, and it must be acknowledged that the risk of misclassification of malnutrition is substantial [16,17,18,19]. These markers are nonspecific, and laboratory reference ranges do not necessarily reflect underlying metabolic disequilibrium. Therefore, it is increasingly recommended to integrate information from all available methods in a given case to reliably identify patients for whom nutritional intervention is essential [20].

All these problems, as in a lens, focus on an extremely specific group of patients, namely heart recipients. In patients debilitated by heart disease, in whom the timing of surgery cannot be planned, comprehensive nutritional intervention is challenging to implement due to the complexity of multi-organ interactions and the use of pharmacotherapy, which significantly affects commonly used nutritional markers [21,22].

This study primarily focused on identifying laboratory biomarkers that could facilitate and expedite the detection of malnutrition in patients following orthotopic heart transplantation (OHT). The aim was to investigate the associations between selected routinely measured venous blood parameters during hospitalization and the risk of malnutrition assessed based on BMI.

## 2. Materials and Methods

### 2.1. Study Design, Population, and Data Collection

A retrospective analysis was conducted on the available medical records of 100 randomly selected patients who underwent OHT between 2021 and 2024 at a tertiary-level heart disease centre with a total of over 1000 heart transplants performed (Silesian Centre for Heart Diseases, Zabrze, Poland). Patients receiving renal replacement therapy within the previous six months or those being evaluated for simultaneous heart and kidney transplantation (*n* = 5) were excluded from the study. The Bioethics Committee waived the requirement for informed consent due to the retrospective and non-interventional nature of the project (analysis of medical records) (approval number: PCN/CBN/0052/KB/116/22, Ethics Committee of the Medical University of Silesia, Katowice, 16 May 2022).

**Figure 1:**
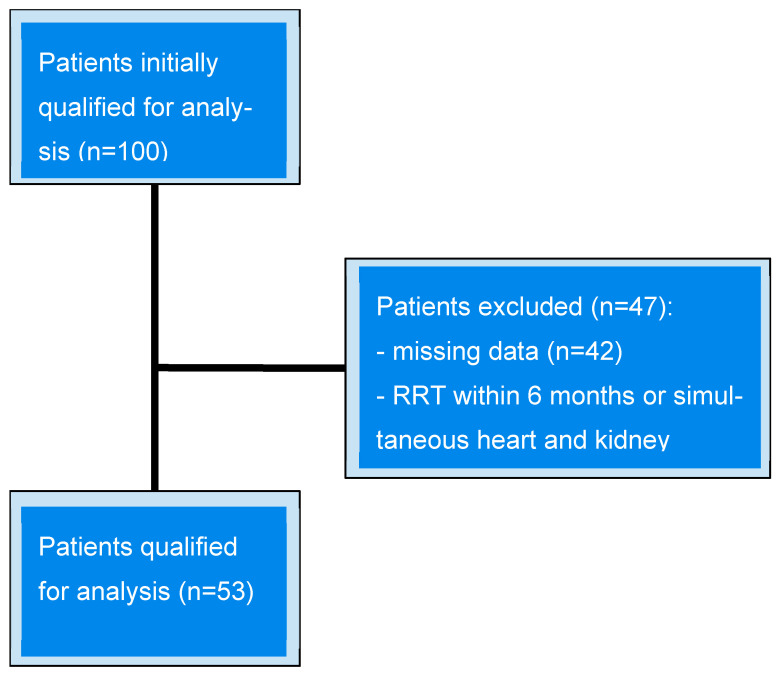
Patient qualification flowchart. RRT—Renal Replacement Therapy.

Patients with missing data for any laboratory variable were excluded from the analysis (*n* = 42). Additionally, patients receiving renal replacement therapy (RRT) within the previous six months or those being evaluated for simultaneous heart and kidney transplantation (*n* = 5) were excluded from the study (Figure 1).

The analysis of available data related to the study aim included gender, age, BMI, and routinely measured laboratory biomarkers (albumin, total serum protein, complete blood count, *C*-reactive protein (CRP), creatinine, blood urea nitrogen), patient comorbidities (ischaemic or non-ischaemic cardiomyopathy, diabetes, chronic kidney disease, chronic lung disease). Additional calculable parameters were also obtained based on available data (Lymphocyte-to-leukocyte ratio, neutrophil-to-lymphocyte). The selection of variables was based on the available literature [23]. Data were obtained from the hospital electronic system AMMS (Asseco, Gdańsk, Poland), considering values from hospital admission up to the heart transplantation procedure. Nutritional status classification was based on recommended reference values from the literature [23], or, if unavailable, on local laboratory standards. BMI was not used as a nutritional marker in the analyses due to its potential confounding by fluid management for chronic heart failure stabilization and numerous concerns regarding its reliability. For BMI analysis, a cut-off value of <22 kg/m^2^ was applied, as recommended by the European Society for Clinical Nutrition and Metabolism (ESPEN) for geriatric or clinically compromised patients, to dichotomize the study population [24]. Age categories were defined dichotomously based on the median age of the evaluated population.

Adequate efforts were made to reduce the risk of potential bias. Patients were enrolled in the study based on a single primary variable, which was heart transplantation, potentially reducing the risk of selection bias. The disqualification of a significant group of patients, which obviously reduced the statistical power, was mainly due to the lack of data (clinical or laboratory) necessary to perform the previously assumed calculations and analyses, and was therefore independent of the researchers. The lack of laboratory test results is mainly due to the lack of a standardized and uniform set of tests that should be ordered during patient care. The decision is made on the basis of the clinical condition and diagnostic needs determined by the attending physician, so it is difficult in this case to talk about any purposefulness in ordering or not ordering laboratory tests—it usually depends on experience and preferences. Thus, the risk of selection bias in this case seems low—the exclusion of such a large group of patients from the study should not affect internal validity. No significant confounding factors were identified that could have significantly affected the analysis of the collected data. The evaluation of patients from a single centre eliminates the risk of measurement errors resulting, for example, from different methods of determining results or the use of different laboratory standards.

### 2.2. Statistical Analysis

Statistical analyses were performed using MedCalc v. 15.4 software. Continuous variables are presented as median and interquartile range (IQR), while categorical variables are expressed as absolute values and percentages. The distribution of variables was assessed using the Shapiro–Wilk test. Between-group differences were analyzed using Student’s *t*-test or the Kruskal–Wallis test, as appropriate. Diagnostic accuracy was evaluated using receiver operating characteristic (ROC) curves. Correlations were assessed using Spearman’s rank correlation coefficient for continuous variables and the contingency coefficient for categorical variables. A significance level of *p* < 0.05 was applied in all analyses.

## 3. Results

The study group consisted of 53 patients, 7 women and 46 men. The median age was 54 years (IQR 44–62). The median BMI was 26.3 kg/m^2^ (IQR 23–29). Eight patients (15%) were undernourished, defined as BMI < 22 kg/m^2^. The characteristics of the subjects, including laboratory parameters of nutritional status, are presented in Table 1.

Only the distribution of albumin, hemoglobin, and total protein values in the evaluated population was normal, while the other parameters deviated from normal. Considering the accepted laboratory standards, the prevalence of malnutrition among the subjects ranged from 13% to 100% (Table 2), which translated into discrepancies in estimating malnutrition using these biomarkers at a level of 87% (relative difference of >500%).

Differences in the values of the analyzed nutritional status parameters and the prevalence of malnutrition according to gender (women vs. men), age (older vs. younger), BMI (<22 vs. ≥22 kg/m^2^) and Nutritional Risk Screening 2002 (NRS-2002) score (<3 vs. ≥3) are presented in Table 3, Table 4, Table 5 and Table 6. Cholesterol levels were significantly higher in women and creatinine and BUN in men. In the other subgroups analyzed (age, BMI, NRS-2002), the values of the studied parameters did not differ significantly.

No significant differences were observed between the analyzed markers in the low- and high-risk malnutrition groups defined according to the NRS 2002 score (Table 6).

**Figure 2:**
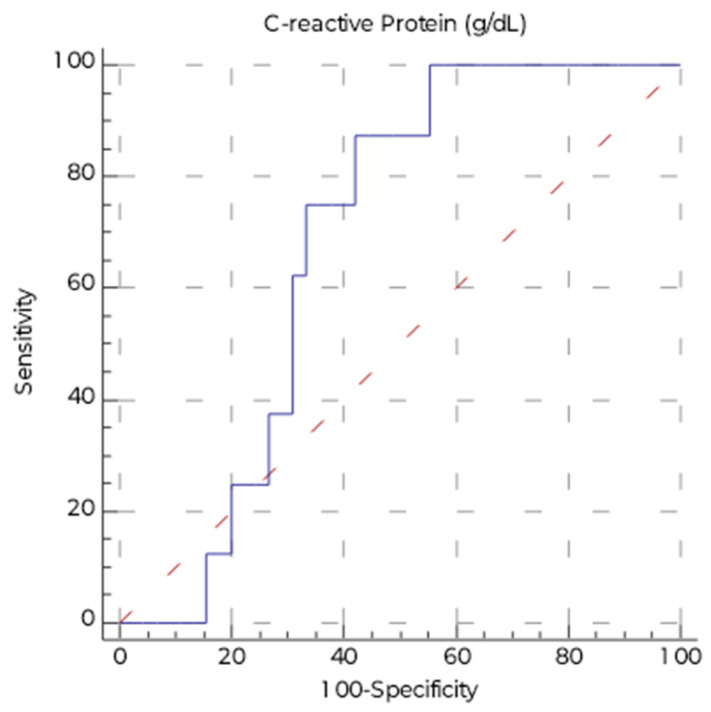
ROC curve for evaluating the diagnostic accuracy of CRP in predicting malnutrition (BMI < 22 kg/m^2^).

Table 7 presents the areas under the ROC curves (AUROCs) for predicting malnutrition based on the studied variables, using the recommended BMI cut-off of 22 kg/m^2^. Only CRP with a weak accuracy predicted the risk of malnutrition (Figure 2), although the CRP value itself did not differ significantly between people with higher and lower BMI (Table 5).

Finally, correlations between the analyzed parameters (Spearman’s rank correlation coefficients) and agreement in malnutrition classification using these variables (contingency coefficient) were evaluated (Table 8; Table 9). Negative correlations were found between CRP and total protein (−0.342; *p* = 0.012), albumin (−0.666; *p* < 0.0001), cholesterol (−0.287; *p* = 0.037), and hemoglobin (−0.383; *p* = 0.0046). A positive correlation was observed between CRP and NLR (0.333; *p* = 0.014). Other statistically significant correlations are highlighted in bold in Table 2.

## 4. Discussion

Malnutrition is an important clinical problem with a significant impact on both hospital and long-term prognosis. It is particularly important in patients with severe chronic heart failure, including those being evaluated for organ transplantation [8]. The effects of malnutrition, metabolic dysfunction and postoperative complications should be considered when developing a nutrition care plan for patients in the immediate post-transplant phase [25]. The aim of this study was to determine which routinely measured laboratory parameters—including composite indices derived from them—can aid in identifying patients with malnutrition or wasting. Patients with severe heart failure develop various forms of frailty syndrome, or rather anorexia-cachexia-asthenia syndrome [26], originally described in the oncology patient population. We have shown that assessing nutritional status and defining malnutrition in patients with severe heart failure who are heart recipients is difficult and should not be based on laboratory parameters classically used in clinical practice. It is necessary to combine several of them or search for new biomarkers to increase the accuracy of diagnosis, which will be particularly important in terms of the possibility of designing reliable studies assessing the impact of nutritional interventions on prognosis. Implementing and adhering to a nutrition support protocol for Orthotopic Heart Transplantation (OHT) patients increases nutrient delivery and is associated with a reduced risk of complications [27].

Numerous studies have focused on the assessment of malnutrition risk in chronic heart disease [28,29,30,31]. Malnutrition is not only unintended weight loss but, more importantly, changes in body composition (with the most detrimental effect from muscle mass loss) and progressive metabolic disequilibrium (including impaired central hormonal regulation). The most commonly cited parameters, either individually or in combination, that may help identify patients at risk include total protein, albumin, prealbumin, total cholesterol, hemoglobin, leukocyte parameters (leukocytes, lymphocytes, NLR), and markers of inflammation (CRP). Various cut-off points for identifying malnutrition risk have been reported depending on the study population or methodology and often differ from standard laboratory reference ranges, as demonstrated in our analysis. BMI is widely used in clinical practice to assess malnutrition and is considered a more reliable tool than individual laboratory measurements. It is also an integral component of validated screening tools such as MNA, MUST, and NRS-2002 [23].For many years, it has been emphasized that BMI is not a reliable indicator, although no superior routine anthropometric assessment method has yet been proposed. The routine cut-offs recommended by the World Health Organization (WHO) do not correspond to the clinical risk of malnutrition or patient survival. The arbitrarily defined “normal” BMI range (18.5–25 kg/m^2^) does not correspond to the clinical norm (22–27.5 kg/m^2^), which is considered the range associated with the most favourable prognosis—a phenomenon also referred to as the “obesity paradox” [32]. Duerksen et al. highlight the importance of physical examination in the assessment and diagnosis of malnutrition. A well-validated bedside tool used for this purpose is the Subjective Global Assessment (SGA), which relies on features obtained from the medical history and physical examination (including loss of subcutaneous fat, muscle wasting, and the presence of edema) and classifies patients on a scale ranging from well-nourished to severely malnourished [33]. The sensitivity and specificity of SGA for diagnosing malnutrition in geriatric patients were reported to be 82% and 72%, respectively [34].

In our study, we demonstrated that only CRP, with weak accuracy, predicted the risk of malnutrition. However, as mentioned, CRP levels can also be influenced by factors unrelated to nutrition (e.g., cardiovascular diseases) and other inflammatory conditions (e.g., infections). Therefore, from a clinical perspective, CRP should be interpreted solely as a complementary marker to a detailed assessment of nutritional status [23]. Similar findings were reported by Mądra-Gackowska et al., who observed that patients classified as being at high risk of malnutrition according to the Geriatric Nutritional Risk Index (GNRI) had a significantly higher median CRP level compared with the other groups. Notably, the group of well-nourished patients also included individuals with the highest CRP values, suggesting that relying solely on *C*-reactive protein concentration to assess nutritional status may lead to misclassification [35]. The results of our study are partly consistent with observations from a meta-analysis evaluating various markers of malnutrition in older adults [23]. That analysis demonstrated that no single parameter is ideal, although among the available markers, BMI, hemoglobin, and total cholesterol were found to be the most useful. In the context of acute illness, BMI, hemoglobin, total cholesterol, and total protein showed better predictive potential than CRP and leukocyte parameters. These negative findings highlight that reliance on single markers alone is inadequate in the population of heart transplant recipients, emphasizing the need for more comprehensive strategies to accurately identify patients at risk of malnutrition. Consequently, future research should aim to identify novel laboratory biomarkers to support the development of integrated models for detecting malnutrition in this population, focusing on approaches that are practical for routine clinical use and incorporate thorough patient assessment.

For this analysis, prealbumin and transferrin were not assessed as potentially more reliable markers of nutritional status, as these tests were not routinely performed during the study period. In heart transplant patients, inflammatory states are associated with an inverse relationship between hepatic protein synthesis and CRP levels, resulting in frequent deficiencies of visceral proteins in those with elevated acute-phase reactants [36]. Prealbumin screening should be performed only after excluding an acute inflammatory state (CRP > 15 mg/L). In patients not admitted to the intensive care unit and without signs of inflammation, prealbumin can serve as a useful prognostic marker of nutritional status. An increase in prealbumin levels of less than 0.04 g/L per week may indicate unsuccessful nutritional therapy [16]. Conversely, other studies have shown that serum visceral proteins (albumin and prealbumin) are not reliable predictors of nutrient deficiencies and should not be used as the sole criterion for guiding nutritional therapy, particularly in cohorts consisting mainly of patients with anorexia nervosa [37]. This may be due to the fact that serum visceral proteins, including albumin and prealbumin, primarily reflect the body’s response to inflammation and disease severity, and are influenced by fluid status rather than nutrient intake alone. According to American Society for Parenteral and Enteral Nutrition (ASPEN) guidelines, they are best considered markers of “nutrition-related risk” associated with inflammation [38]. In the study by Yeh et al., involving adult surgical Intensive Care Unit (ICU) patients receiving enteral nutrition, it was shown that initial serum albumin levels and their changes over time are inversely associated with inflammation. Although baseline albumin levels may reflect the patient’s nutritional status, neither albumin levels nor prealbumin trends correlate with calorie or protein deficits and should not be used to assess the adequacy of nutritional support [39]. To add to this, low serum albumin levels may be associated with HF. Albumin plays a key role in maintaining oncotic pressure and modulating the body’s antioxidant and anti-inflammatory responses. In patients with heart failure, hypoalbuminemia promotes fluid shift into the extravascular space, exacerbating fluid retention and edema, and has also been associated with myocardial fibrosis. Reduced ejection fraction in advanced heart failure leads to organ hypoperfusion and activation of the renin–angiotensin–aldosterone system, further accelerating disease progression [10,39]. Consequently, heart failure and malnutrition mutually reinforce each other, resulting in poorer prognosis. Interestingly, hypoalbuminemia appears to be particularly strongly associated with heart failure with preserved ejection fraction. This association may be mediated by chronic inflammation, which contributes to myocardial diastolic dysfunction, whereas inhibition of inflammatory pathways has been shown to improve cardiac function in experimental models [40]. Regarding transferrin, the literature presents conflicting evidence on its utility in assessing nutritional status. Although serum transferrin levels decrease in cases of severe malnutrition, it has been shown to be an unreliable marker for mild malnutrition and for evaluating lean body mass in older patients [16,41].

In a cohort of 60 patients assessed five years after OHT, Prenner et al. found that only serum creatinine (AUROC = 0.698) and albumin (AUROC = 0.606) were statistically significant predictors of malnutrition, in contrast to BMI (AUROC = 0.515) [18]. The nutrition risk index (NRI) was found to be independently associated with a risk of postoperative infection (OR= 0.97) and prolonged postoperative ventilator support (OR = 0.96) [7]. In the study by Almutawa et al., participants with higher post–heart transplant NRI had an 18% lower risk of death compared with those with lower post-transplant NRI (HR = 0.82; 95% CI, 0.75–0.89; *p* < 0.001) [9]. Similarly, another study demonstrated a significant independent association between lower pre–heart transplant NRI and shorter post-transplant survival [42]. Interestingly, NRI may be affected by gender [9]. In a pilot study in Italian heart transplant recipients it was concluded that malnutrition assessed with the MUST score seems not to be associated with short-term mortality or major post-operative complications, but may affect long-term mortality [22], which is probably related with a plethora of co-variates which affect short-term prognosis. Bayram et al., in a cohort of 195 patients after OHT with a median follow-up of 503.5 days, demonstrated that malnutrition defined by a CONUT score ≥ 2 was an independent predictor of mortality, whereas malnutrition defined by the Prognostic Nutritional Index (PNI) was not [43]. Yoo and co-workers found that GNRI, PNI and CONUT at time of HT operation were not associated with subsequent mortality but showed prognostic utility only at time of HT listing in those with severe heart failure [20]. In turn, in lung transplant recipients, preoperative albumin levels < 3.5 g/dL (HR = 2.723) and hemoglobin < 13 g/dL were independent predictors of death [44].

All above-mentioned studies underline the need for further investigations on the role of available biomarkers and potential confounders. Considering the widespread use of laboratory biomarkers, our findings add valuable insight into their utility in detecting malnutrition in OHT patients in everyday clinical practice.

## 5. Study Limitations

Firstly, our study is a retrospective, single-centre study with a risk of selection bias. The relatively small sample size (53 patients), resulting from the exclusion of a substantial number of individuals with missing data, reduces statistical power, increases the risk of type II error, and may introduce additional selection bias. This approach was deliberately implemented to ensure methodological rigour and the reliability of the analysis. As the investigation was designed as a pilot study, the obtained results provide a basis for planning a future prospective study in a larger patient population. It also seems that risk of potential selection bias is low. The heterogeneity in admission indications, comorbidities, nutritional status, as well as the duration and stage of disease among the included patients, limits the generalizability of the findings to the broader population of hospitalized individuals. Additionally, potential confounding factors—such as comorbidities and pharmacotherapy—were not sufficiently controlled for in the analysis, which may have influenced the interpretation of the associations between laboratory parameters and nutritional status. In our planned prospective study involving a larger patient cohort, we will perform a stratified analysis to account for comorbidities and medication use, using the Mantel–Haenszel method. The statistical analysis highlights the need to replicate this study in a larger cohort to more accurately identify trends for individual laboratory parameters and to determine the sensitivity and specificity of the cut-off values in the population of heart transplant recipients. Secondly, CRP showed limited predictive value for identifying malnutrition. Its levels are highly influenced by factors such as inflammation, which substantially limits its usefulness as an independent marker for assessing nutritional status in heart transplant recipients. Therefore, we believe CRP should be interpreted with caution, and contemporary nutritional indices—such as the CRP-to-albumin ratio (CAR) or the CRP-to-prealbumin ratio (CPR)—should be incorporated into the assessment of malnutrition in critically ill patients. Third, we did not assess prealbumin and transferrin concentrations as more reliable parameters of nutritional status than albumin and total protein, as they were not routinely ordered between 2021 and 2024, and their wider implementation occurred only recently, which may have limited their use in the analysis. The BMI threshold adopted in this study served only as a reference point rather than a definitive diagnostic criterion. However, while it has reference value, it does not incorporate more precise and objective measures, such as body composition analysis, muscle mass assessment using ultrasound or CT imaging and bioelectrical impedance analysis [45]. Finally, we did not take into account tools such as questionnaires or indices: SGA, MUST, MST, NRI, NSI or MNA-SF. Although this was intentional, we preferred to focus on a comprehensive statistical analysis of the various laboratory markers available at our centre. Finally, the study did not include follow-up data after nutritional intervention in heart transplant recipients, preventing assessment of temporal changes and the practical utility of the indicators in monitoring intervention effects. However, including an analysis of dynamic changes could affect the representativeness of the results. Therefore, future studies should include longitudinal follow-up and we recommend validation of our results in a prospective study.

## 6. Conclusions

Assessment of nutritional status and the diagnosis of malnutrition in patients with advanced heart failure who are heart transplant recipients is challenging and should not rely solely on conventional laboratory parameters. Early recognition and timely intervention for malnutrition may significantly improve outcomes in these patients. Clinicians should consider that isolated deviations in individual laboratory parameters are insufficient for a definitive diagnosis of malnutrition. When such deviations are observed, it is difficult to determine whether they result from comorbid conditions, the underlying disease, medications or supplements, or from malnutrition itself. Combining multiple markers or identifying new biomarkers is essential to enhance diagnostic accuracy, which is particularly important when designing robust studies to evaluate the impact of nutritional interventions on patient outcomes.

## Figures and Tables

**Table 1:** Characteristics of the study population.

Variable	Value
Gender (F/M)	7/46 (13/87)
Age (years)	54 (44–62)
BMI (kg/m^2^)	26.3 (23–29)
Ischaemic cardiomyopathy	22 (42)
Non-ischaemic cardiomyopathy	31 (58)
Diabetes	16 (30)
Chronic kidney disease	16 (30)
Chronic lung disease	3 (5)
Albumin (g/dL)	40 (36.75–43.25)
Total protein (g/dL)	67 (62–71.25)
Total cholesterol (mg/dL)	3.44 (2.64–4.13)
Leukocytes (10^3^/mm^3^)	17.51 (11.53–21.43)
Lymphocytes (10^3^/mm^3^)	1.39 (1.1–2.19)
Lymphocyte-to-leukocyte ratio	0.09 (0.06–0.12)
Neutrophil-to-lymphocyte ratio	3.54 (1.98–5.32)
Hemoglobin (g/dL)	11.6 (9.99–13.87)
Hematocrit (%)	31.60 (28.87–36.50)
*C*-Reactive Protein (g/dL)	8.95 (2.99–22.17)
Creatinine (mmol/L)	110 (82.75–132.75)
Blood UreaNitrogen (mmol/L)	4.57 (3.45–6.95)

Continuous variables are presented as median and IQR (in parentheses), and categorical variables as absolute values and percentages (in parentheses).

**Table 2:** Prevalence of malnutrition based on the analyzed laboratory parameters.

Laboratory Parameter	Suggested Cut-Off for Malnutrition Assessment	Percentage of Individuals with Malnutrition
Albumin (g/dL)	<3.5	7 (13.2%)
Total protein (g/dL)	<6	8 (15.1%)
Total cholesterol (mmol/L)	<8.89	53 (100%)
Lymphocytes (10^3^/mm^3^)	<1 or >3	12 (22.6%)
Hemoglobin (g/dL)	<13	35 (66%)
*C*-Reactive Protein (g/dL)	>10	24 (45.3%)
Creatinine (mmol/L)	<62	15 (28.3%)
Blood UreaNitrogen (mg/dL)	<7	40 (75.5%)

Categorical variables are presented as absolute values and percentages (in parentheses).

**Table 3:** Analyzed laboratory parameters stratified by gender.

Laboratory Parameter	Women	Men	*p*
Albumin (g/dL) (N)	38 (35.22–40.77)	40.5 (38.96–42.07)	0.222
Total protein (g/dL) (N)	63.71 (58.77–68.65)	67.84 (65.52–70.17)	0.184
Total cholesterol (mg/dL)	4.62 (3.45–5.60)	3.35 (2.95–3.59)	0.022
Leukocytes (10^3^/mm^3^)	17.56 (9.42–29.34)	17.09 (14.90–19.1)	0.545
Lymphocytes (10^3^/mm^3^)	2.66 (1.05–27.34)	1.32 (1.17–1.58)	0.043
Lymphocyte-to-leukocyte ratio	0.11 (0.04–2.58)	0.09 (0.07–0.10)	0.441
Neutrophil-to-lymphocyte ratio	1.87 (0.54–8.25)	3.64 (2.95–4.65)	0.171
Hemoglobin (g/dL) (N)	10.44 (8.56–12.33)	12.02 (11.31–12.74)	0.717
Hematocrit (%)	31.60 (27.44–40.38)	31.55 (29.86–34.05)	0.958
*C*-Reactive Protein (g/dL)	13.47 (0.72–80.21)	8.93 (4.44–12.07)	0.926
Creatinine (mmol/L)	78 (43–101.08)	115 (105.88–125.1)	0.001
Blood Urea Nitrogen	3.84 (1.75–4.50)	4.70 (3.99–6.24)	0.023

The variables are presented as median or mean (depending on test type) and 95% CI for median or mean, and statistical significance of intergroup differences ‘*p*’ (result of t-Student or U-Mann–Whitney test).

**Table 4:** Analyzed laboratory parameters stratified by age.

Laboratory Parameter	Younger (<54)	Older (≥54)	*p*
Albumin (g/dL) (N)	41.58 (39.35–43.81)	39.03 (37.26–40.8)	0.067
Total protein (g/dL) (N)	68.5 (65.33–71.66)	66.31 (63.36–69.25)	0.303
Total cholesterol (mg/dL)	3.41 (2.91–3.97)	3.45 (3.01–4.04)	0.886
Leukocytes (10^3^/mm^3^)	18.77 (15.69–21.65)	16.06 (12.70–17.73)	0.086
Lymphocytes (10^3^/mm^3^)	1.26 (1.15–1.69)	1.40 (1.20–2.14)	0.480
Lymphocyte-to-leukocyte ratio	0.08 (0.06–0.10)	0.10 (0.07–0.13)	0.223
Neutrophil-to-lymphocyte ratio	3.40 (2.46–4.68)	3.72 (1.98–5.24)	0.900
Hemoglobin(g/dL) (N)	11.78 (10.71–12.86)	11.84 (10.95–12.73)	0.933
Hematocrit (%)	31.10 (28.47–34.12)	31.60 (29.95–35.35)	0.312
*C*-Reactive Protein (g/dL)	9.28 (2.79–23.89)	8.92 (3.87–13.26)	0.928
Creatinine (mmol/L)	111.5 (90.21–120.77)	110.1 (98.12–125.2)	0.816
Blood Urea Nitrogen	4.27 (3.56–5.53)	4.63 (3.98–6.27)	0.549

The variables are presented as median or mean (depending on test type) and 95% CI for median or mean, and statistical significance of intergroup differences ‘*p*’ (result of t-Student or U-Mann–Whitney test). N—normal.

**Table 5:** Analyzed laboratory parameters stratified by BMI.

Laboratory Parameter	BMI < 22	BMI 22+	*p*
Albumin (g/dL) (N)	38.37 (35.88–40.86)	40.51 (38.91–42.10)	0.274
Total protein (g/dL) (N)	65.87 (60.77–70.97)	67.55 (65.17–69.93)	0.571
Total cholesterol (mg/dL)	2.95 (2.57–4.26)	3.45 (3.09–3.95)	0.502
Leukocytes (10^3^/mm^3^)	16.37 (11.80–23.09)	17.56 (14.64–19.23)	0.881
Lymphocytes (10^3^/mm^3^)	1.43 (1.03–2.52)	1.39 (1.21–1.75)	0.663
Lymphocyte-to-leukocyte ratio	0.10 (0.06–0.14)	0.09 (0.07–0.1)	0.447
Neutrophil-to-lymphocyte ratio	4.38 (1.97–9.36)	3.48 (2.46–4.32)	0.285
Hemoglobin (g/dL) (N)	11.34 (10.14–12.53)	11.90 (11.13–12.67)	0.547
Hematocrit (%)	32.9 (26.84–35.29)	31.5 (29.73–34.22)	0.584
*C*-Reactive Protein (g/dL)	13.04 (8.18–42.39)	7.11 (3.37–10.32)	0.106
Creatinine (mmol/L)	105 (58.97–172.82)	110 (100.9–118.53)	0.813
Blood Urea Nitrogen	8.14 (2.55–12.47)	4.37 (3.85–4.83)	0.075

The variables are presented as median or mean (depending on test type) and 95% CI for median or mean, and statistical significance of intergroup differences ‘*p*’ (result of t-Student or U-Mann-Whitney test). N—normal.

**Table 6:** Analyzed laboratory parameters stratified by NRS score.

Laboratory Parameter	NRS < 3	NRS ≥ 3	*p*
Albumin (g/dL) (N)	41 (38.60–43.40)	40.74 (39.30–42.18)	0.863
Total protein (g/dL) (N)	70.33 (64.92–75.74)	67.00 (64.43–69.56)	0.234
Total cholesterol (mg/dL)	3.57 (2.20–4.10)	3.43 (3.05–3.96)	0.930
Leukocytes (10^3^/mm^3^)	17.51 (15.12–23.92)	18.24 (15.29–20.11)	0.782
Lymphocytes (10^3^/mm^3^)	2.01 (1.19–2.55)	1.34 (1.18–1.61)	0.130
Lymphocyte-to-leukocyte ratio	0.09 (0.07–0.14)	0.08 (0.06–0.10)	0.343
Neutrophil-to-lymphocyte ratio	2.45 (1.84–5.23)	3.68 (2.91–4.62)	0.262
Hemoglobin (g/dL) (N)	13.05 (11.83–14.28)	11.89 (11.06–12.72)	0.180
Hematocrit (%)	33.20 (28.20–37.11)	31.90 (29.27–34.97)	0.727
*C*-Reactive Protein (g/dL)	8.92 (1.44–12.30)	7.50 (3.35–13.11)	0.976
Creatinine (mmol/L)	102 (74.82–138.06)	110 (90.72–119.51)	0.930
Blood Urea Nitrogen	3.99 (3.41–6.91)	4.63 (4.15–5.10)	0.631

The variables are presented as median or mean (depending on test type) and 95% CI for median or mean, and statistical significance of intergroup differences ‘*p*’ (result of t-Student or U-Mann-Whitney test).

**Table 7:** Diagnostic accuracy of individual laboratory parameters for detecting malnutrition.

Laboratory Parameter	AUROC (95% CI)	*p*
Albumin	0.650 (0.507–0.776)	0.085
Total protein	0.581 (0.437–0.715)	0.411
Total cholesterol	0.485 (0.432–0.710)	0.485
Lymphocytes	0.549 (0.406–0.686)	0.689
Hemoglobin	0.565 (0.422–0.701)	0.449
*C*-Reactive Protein	0.681 (0.538–0.802)	0.014
Creatinine	0.526 (0.385–0.665)	0.843
Blood Urea Nitrogen	0.699 (0.557–0.817)	0.150

Variables are presented as the area under the ROC curve (AUC, 95% CI) along with the statistical significance (*p*-value). AUROC—Area Under the Receiver Operating Characteristic curve.

**Table 8:** Correlation between laboratory parameter values (continuous variables).

	ALB	TP	CHOL	LEU	LYMPH	LLR	NLR	HGB	HCT	CRP	CREA	BUN
ALB	-	0.679 (<0.0001)	0.300 (0.029)	0.121 (0.386)	0.121 (0.389)	0.047 (0.736)	−0.380 (0.005)	0.497 (0.0002)	0.164 (0.241)	−0.666 (<0.0001)	0.030 (0.827)	−0.130 (0.352)
TP	0.679 (<0.0001)	-	0.293 (0.033)	0.099 (0.476)	0.226 (0.104)	0.145 (0.299)	−0.335 (0.014)	0.290 (0.035)	0.134 (0.337)	−0.342 (0.012)	0.001 (0.988)	−0.101 (0.472)
CHOL	0.300 (0.029)	0.293 (0.033)	-	0.125 (0.372)	0.08 (0.554)	0.004 (0.974)	−0.122 (0.384)	0.229 (0.099)	0.046 (0.740)	−0.287 (0.037)	−0.194 (0.164)	−0.155 (0.267)
LEU	0.121 (0.386)	0.099 (0.476)	0.125 (0.372)	-	0.139 (0.320)	−0.594 (<0.0001)	0.014 (0.915)	0.103 (0.464)	−0.090 (0.520)	−0.068 (0.625)	−0.159 (0.255)	−0.132 (0.347)
LYMPH	0.121 (0.389)	0.226 (0.104)	0.08 (0.554)	0.139 (0.320)	-	0.651 (<0.0001)	0.718 (<0.0001)	0.102 (0.465)	0.202 (0.147)	−0.243 (0.079)	−0.197 (0.157)	−0.265 (0.055)
LLR	0.047 (0.736)	0.145 (0.299)	0.004 (0.974)	−0.594 (<0.0001)	0.651 (<0.0001)	-	−0.538 (<0.0001)	0.027 (0.847)	0.268 (0.052)	−0.141 (0.313)	−0.025 (0.857)	−0.065 (0.643)
NLR	−0.380 (0.005)	−0.335 (0.014)	−0.122 (0.384)	0.014 (0.915)	−0.718 (<0.0001)	−0.538 (<0.0001)	-	−0.240 (0.082)	−0.180 (0.198)	0.333 (0.014)	0.249 (0.072)	0.397 (0.003)
HGB	0.497 (0.0002)	0.290 (0.035)	0.229 (0.099)	0.103 (0.464)	0.102 (0.465)	0.027 (0.847)	−0.240 (0.082)	-	0.264 (0.056)	−0.383 (0.0046)	0.063 (0.649)	−0.050 (0.721)
HCT	0.164 (0.241)	0.134 (0.337)	0.046 (0.740)	−0.090 (0.520)	0.202 (0.147)	0.268 (0.052)	−0.180 (0.198)	0.264 (0.056)	-	−0.263 (0.057)	0.102 (0.466)	0.164 (0.241)
CRP	−0.666 (<0.0001)	−0.342 (0.012)	−0.287 (0.037)	−0.068 (0.625)	−0.243 (0.079)	−0.141 (0.313)	0.333 (0.014)	−0.383 (0.0046)	−0.263 (0.057)	-	−0.013 (0.923)	0.205 (0.140)
CREA	0.030 (0.827)	0.001 (0.988)	−0.194 (0.164)	−0.159 (0.255)	−0.197 (0.157)	−0.025 (0.857)	0.249 (0.072)	0.063 (0.649)	0.102 (0.466)	−0.013 (0.923)	-	0.449 (0.0008)
BUN	−0.130 (0.352)	−0.101 (0.472)	−0.155 (0.267)	−0.132 (0.347)	−0.265 (0.055)	−0.065 (0.643)	0.397 (0.003)	−0.050 (0.721)	0.164 (0.241)	0.205 (0.140)	0.449 (0.0008)	-

Spearman’s rank correlation coefficient and its statistical significance (*p*-value in parentheses). ALB—Albumin; BUN—Blood Urea Nitrogen; CHOL—Cholesterol; CREA—Creatinine; CRP—*C*-Reactive Protein; HCT—Hematocrit; HGB—Hemoglobin; LEU—Leukocytes; LLR—Lymphocyte-to-Leukocyte Ratio; LYMPH—Lymphocytes; NLR—Neutrophil-to-Lymphocyte Ratio; TP—Total Protein.

**Table 9:** Correlation between the presence of malnutrition defined by laboratory parameters (categorical variables, based on cut-off points from Table 2).

	ALB	TP	CHOL	LYMPH	HGB	CRP	CREA	BUN
ALB	-	0.356 (0.005)	0.583 (<0.0001)	0.362 (0.004)	0.216 (0.107)	0.349 (0.006)	0.006 (0.965)	0.028 (0.837)
TP	0.356 (0.005)	-	0.562 (<0.0001)	0.321 (0.013)	0.240 (0.072)	0.092 (0.498)	0.021 (0.880)	0.066 (0.631)
CHOL	0.583 (<0.0001)	0.562 (<0.0001)	-	0.467 (0.0001)	0.289 (0.028)	0.075 (0.582)	0.639 (<0.0001)	0.440 (0.0004)
LYMPH	0.362 (0.004)	0.321 (0.013)	0.467 (0.0001)	-	0.238 (0.074)	0.184 (0.173)	0.069 (0.614)	0.058 (0.671)
HGB	0.216 (0.107)	0.240 (0.072)	0.289 (0.028)	0.238 (0.074)	-	0.208 (0.122)	0.021 (0.876)	0.084 (0.537)
CRP	0.349 (0.006)	0.092 (0.498)	0.075 (0.582)	0.184 (0.173)	0.208 (0.122)	-	0.098 (0.471)	0.054 (0.694)
CREA	0.006 (0.965)	0.021 (0.880)	0.639 (<0.0001)	0.069 (0.614)	0.021 (0.876)	0.098 (0.471)	-	0.080 (0.560)
BUN	0.028 (0.837)	0.066 (0.631)	0.440 (0.0004)	0.058 (0.671)	0.084 (0.537)	0.054 (0.694)	0.080 (0.560)	-

Contingency coefficient and its statistical significance (*p*-value in parentheses). ALB—Albumin; BUN—Blood Urea Nitrogen; CHOL—Cholesterol; CREA—Creatinine; CRP—*C*-Reactive Protein; HGB—Hemoglobin; LYMPH—Lymphocytes; TP—Total Protein.

## Data Availability

The data presented in this study are available on request from the corresponding author due to data privacy and legal issues.

## Abbreviations

The following abbreviations are used in this manuscript:

ASA-PS	American Society of Anaesthesiologists Physical Status
ASPEN	American Society for Parenteral and Enteral Nutrition guidelines
BMI	Body mass index
BUN	Blood Urea Nitrogen
CAR	CRP-to-albumin ratio
CPR	CRP-to-prealbumin ratio
CRP	*C*-reactive protein
CVD	Cardiovascular diseases
ESPEN	European Society for Clinical Nutrition and Metabolism
GNRI	Geriatric Nutritional Risk Index
HF	Heart failure
ICU	Intensive Care Unit
NLR	Neutrophil-to-Lymphocyte Ratio
NRS	Nutritional Risk Screening 2002 scale
OHT	Orthotopic Heart Transplantation
PNI	Prognostic Nutritional Index
RRT	Renal Replacement Therapy
SGA	Subjective Global Assessment
WHO	World Health Organization
